# TricValve System Implantation in Patients With Cardiac Implantable Electronic Devices: A Multicentre Observational Study

**DOI:** 10.1093/ejcts/ezag224

**Published:** 2026-09-15

**Authors:** Kira Osipenko, Iuliana Coti, Paul Werner, Amila Kahrovic, Luis Nombela Franco, Ignacio J Amat Santos, Amir A Mahabadi, Christian Frerker, Federico De Marco, Jan Althoff, Antonio Mangieri, Javier Martín Moreiras, Nikola Pavlović, Joachim Schofer, Ulrich Schäfer, Eike Tigges, Christoph Wilde, Jörg Hausleiter, Omar Abdul-Jawad Altisent, Maurizio Taramasso, Luis Fernando Pajín Valbuena, Joško Bulum, Santiago Ordoñez, Martin Swaans, João Silva Marques, Boris Dickmann, Birgit Zirngast, Daniel Zimpfer, Martin Andreas

**Affiliations:** Department of Cardiac and Thoracic Aortic Surgery, Medical University of Vienna, 1090 Vienna, Austria; Department of Cardiac and Thoracic Aortic Surgery, Medical University of Vienna, 1090 Vienna, Austria; Department of Cardiac and Thoracic Aortic Surgery, Medical University of Vienna, 1090 Vienna, Austria; Department of Cardiac and Thoracic Aortic Surgery, Medical University of Vienna, 1090 Vienna, Austria; Instituto Cardiovascular, Hospital Clínico San Carlos, Instituto de Investigación Sanitaria del Hospital Clínico San Carlos (IdISSC), 28040 Madrid, Spain; Cardiology Department, Hospital Clínico Universitario de Valladolid, 47003 Valladolid, Spain; Department of Cardiology and Vascular Medicine, West German Heart and Vascular Center Essen, University Hospital Essen, University of Duisburg-Essen, 45147 Essen, Germany; University Heart Center Lübeck, Medical Clinic II (Cardiology/Angiology/Intensive Care Medicine) and German Center for Cardiovascular Research (DZHK), Partner Site North, 23538 Lübeck, Germany; Centro Cardiologico Monzino IRCCS, 20138 Milan, Italy; Clinic III for Internal Medicine, Faculty of Medicine and University Hospital Cologne, University of Cologne, 50937 Cologne, Germany; IRCCS Humanitas Research Hospital, 20089 Rozzano, Milan, Italy; Cardiology Department, Complejo Asistencial Universitario de Salamanca (CAUSA), Universidad de Salamanca (USAL), 37007 Salamanca, Spain; Department of Cardiovascular Diseases, University Hospital Dubrava, 10000 Zagreb, Croatia; Medical Care Center Prof. Mathey, Prof. Schofer, Asklepios Hospital St. Georg, 20099 Hamburg, Germany; Heart Center Bad Bevensen, 29549 Bad Bevensen, Germany; Department of Cardiology and Critical Care Medicine, Asklepios Clinic St. Georg, 20099 Hamburg, Germany; Asklepios Hospital Nord, 22417 Hamburg, Germany; Department of Medicine I, LMU University Hospital, LMU Munich, 81377 Munich, Germany; Hospital Clínic Barcelona, 08036 Barcelona, Spain; Clinic of Cardiac Surgery, HerzZentrum Hirslanden Zurich, 8008 Zurich, Switzerland; University Hospital of Toledo, 45007 Toledo, Spain; University Hospital Centre Zagreb, 10000 Zagreb, Croatia; Department of Cardiology, Instituto Cardiovascular de Buenos Aires (ICBA), 1428 Buenos Aires, Argentina; Department of Cardiology, St. Antonius Hospital, 3435 CM, Nieuwegein, The Netherlands; Department of Cardiology, Hospital de Santa Maria, 1649-028 Lisbon, Portugal; Department of Cardiology, Marien Hospital Witten, 58452 Witten, Germany; Department of Cardiac Surgery, Medical University of Graz, 8036 Graz, Austria; Department of Cardiac and Thoracic Aortic Surgery, Medical University of Vienna, 1090 Vienna, Austria; Department of Cardiac Surgery, Medical University of Graz, 8036 Graz, Austria

**Keywords:** TricValve system, tricuspid regurgitation, cardiac implantable electronic devices, pacemaker leads, transcatheter tricuspid valve intervention, lead-related complications

## Abstract

**Objectives:**

Tricuspid regurgitation (TR) is associated with high morbidity and mortality. The TricValve bicaval valve system has emerged as a transcatheter option for patients with severe symptomatic TR who are not candidates for surgery or orthotopic interventions. However, its safety in patients with preexisting cardiac implantable electronic devices (CIEDs) remains uncertain due to potential lead-related complications. This study aims to evaluate the feasibility and safety of TricValve implantation in patients with preexisting CIED, with particular focus on lead-related complications and the need for lead extraction or repositioning.

**Methods:**

We conducted a multicentre retrospective study including 65 patients with pre-implanted CIED who underwent TricValve implantation between May 2023 and November 2024 at 23 international centres. Pre-procedural computed tomography and echocardiography were analysed to assess device interaction, lead positioning, and complications. The primary end-point was procedural safety, including lead-related events and the need for lead extraction or repositioning during intervention. Secondary end-points included echocardiographic outcomes, CIED/non-CIED-related complications, and survival during follow-up of 159 days (interquartile range [IQR] 60-358 days).

**Results:**

TricValve implantation was technically successful in all patients (100%). Periprocedurally, no lead extraction or repositioning was required. Lead-related complications occurred in 4 patients (6.2%). No procedural deaths or TricValve malfunctions were observed. Post-implantation imaging confirmed stable lead positioning in all remaining cases.

**Conclusions:**

TricValve implantation in patients with preexisting CIED appears feasible. Lead-related complications during early follow-up were rare, manageable, and did not compromise device function or procedural success. Our data do not support routine prophylactic lead extraction before TricValve implantation.

## Graphical abstract

**Figure:**
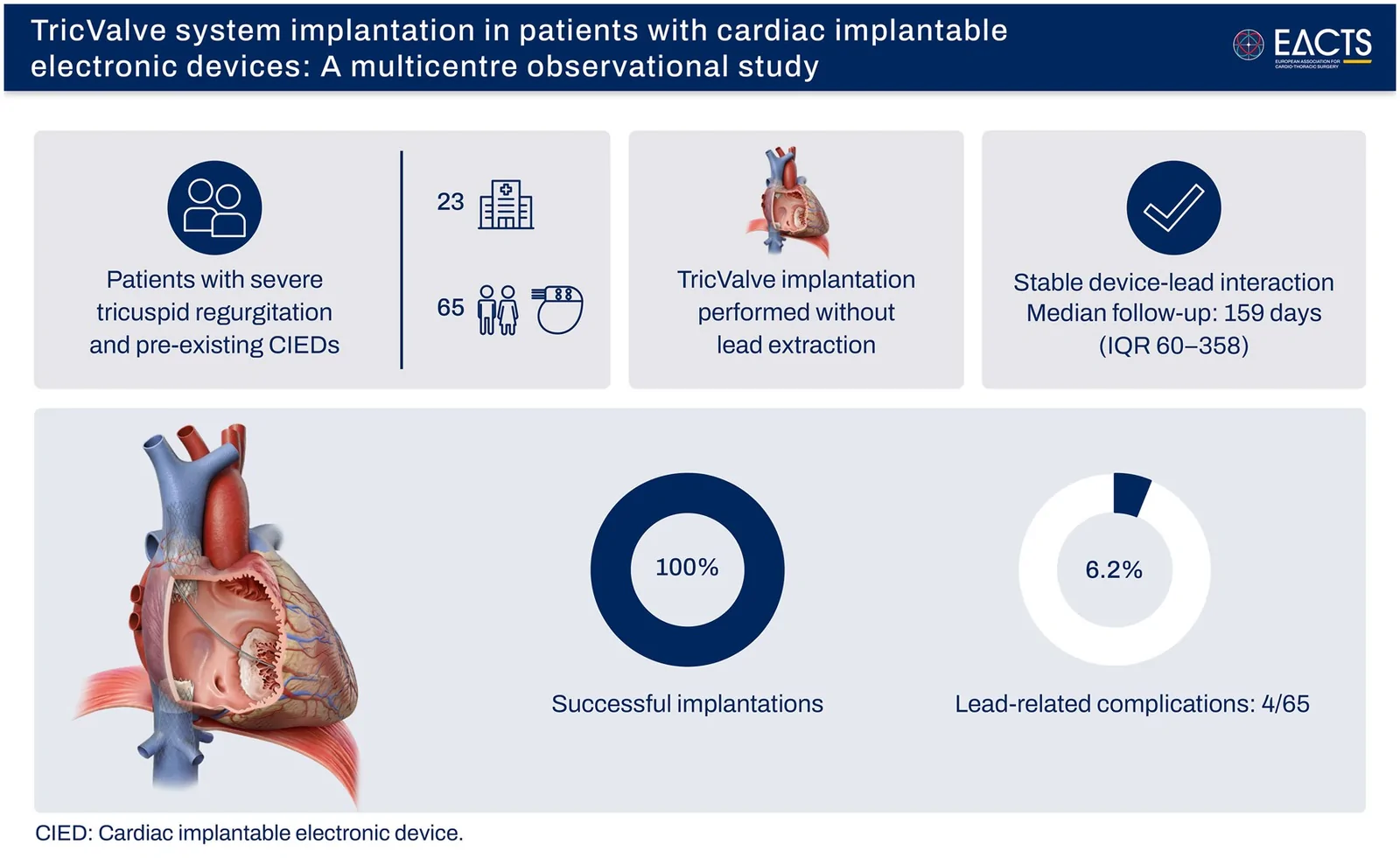

## Introduction

Severe tricuspid valve regurgitation (TR) represents an advanced form of valvular heart disease that is associated with increased morbidity and a significant decline in quality of life.^1^^,^^2^ Currently, several therapeutic options are available for the management of severe TR, including optimal medical therapy, catheter-based interventions, and open-heart surgery.^3^ The choice of treatment depends on the patient’s clinical presentation, comorbidities, and the anatomical characteristics of the tricuspid valve.

In high-risk patients who are not suitable candidates for surgical valve replacement due to elevated operative mortality, transcatheter tricuspid valve replacement (TTVR) with EVOQUE and bicaval valve implantation using the TricValve system have demonstrated promising postprocedural outcomes in patients with severe TR.^4–6^ However, a substantial proportion of patients with progressive TR and heart failure have cardiac implantable electronic devices (CIEDs), which may pose procedural challenges. These devices carry the risk of lead displacement during implantation, potentially resulting in complications such as lead fracture, insulation breach, and impaired sensing or pacing function over time.^7^

The mechanisms of potential lead-related complications may differ depending on the transcatheter treatment strategy. In orthotopic TTVR, transvenous leads may become “jailed” at the level of the native tricuspid annulus, whereas the bicaval TricValve system may interact with pacing leads during deployment of the superior vena cava (SVC) prosthesis. Such interactions may theoretically result in transient lead displacement or altered device function, providing the rationale for evaluating lead-related complications following TricValve implantation.

While initial data on TTVR implantation in the presence of existing CIED leads have already been published, demonstrating that the procedure is feasible and associated with low rates of lead-related complications, comparable data for bicaval valve implantation using the TricValve system are currently lacking.

This raises the important question of whether patients with previously implanted CIEDs may also develop lead-related complications after TricValve implantation, and whether a preprocedural lead extraction is required—an intervention associated with a risk of tricuspid valve injury, including leaflet tears, chordal injury, and papillary muscle rupture, which may result in new or worsening TR reported in approximately 10%-12% of patients undergoing transvenous lead extraction (TLE).^8^^,^^9^

In the absence of extensive studies addressing these concerns, the aim of this multicentre, retrospective study was to evaluate the safety of TricValve system implantation in patients who have previously undergone CIED implantation.

## Methods

### Study design, data collection, and ethical statement

This retrospective, multicentre study was conducted at the Medical University of Vienna and included 65 patients with pre-implanted CIED who underwent TricValve implantation between May 2023 and November 2024 at 23 international centres.

The primary objectives were to evaluate procedural safety, including the feasibility of TricValve implantation in the presence of CIED leads, the occurrence of lead-related events during implantation, and the need for lead extraction or repositioning prior to the procedure (**Figure 1**). Specifically, all centres were asked to report predefined clinically relevant lead-related complications, including lead fracture, lead dislodgement, clinically significant conduction disturbances or device malfunction, the need for lead extraction, lead revision or reimplantation, as well as non-lead-related complications. As all patients underwent routine device follow-up according to local clinical practice, clinically relevant CIED dysfunction requiring intervention would have been captured. The secondary objectives included computed tomography (CT)-based assessment of lead position before the procedure, echocardiographic outcomes, CIED/non-CIED-related complications, and survival during follow-up. To address these objectives, 23 participating centres, comprising 65 patients, were invited to provide detailed data on baseline patient characteristics and the earliest available follow-up. As the TricValve system has been introduced only recently, follow-up was restricted to the maximum duration currently available, with a median follow-up of 159 days (interquartile range [IQR] 60-358 days).

**Figure 1.**
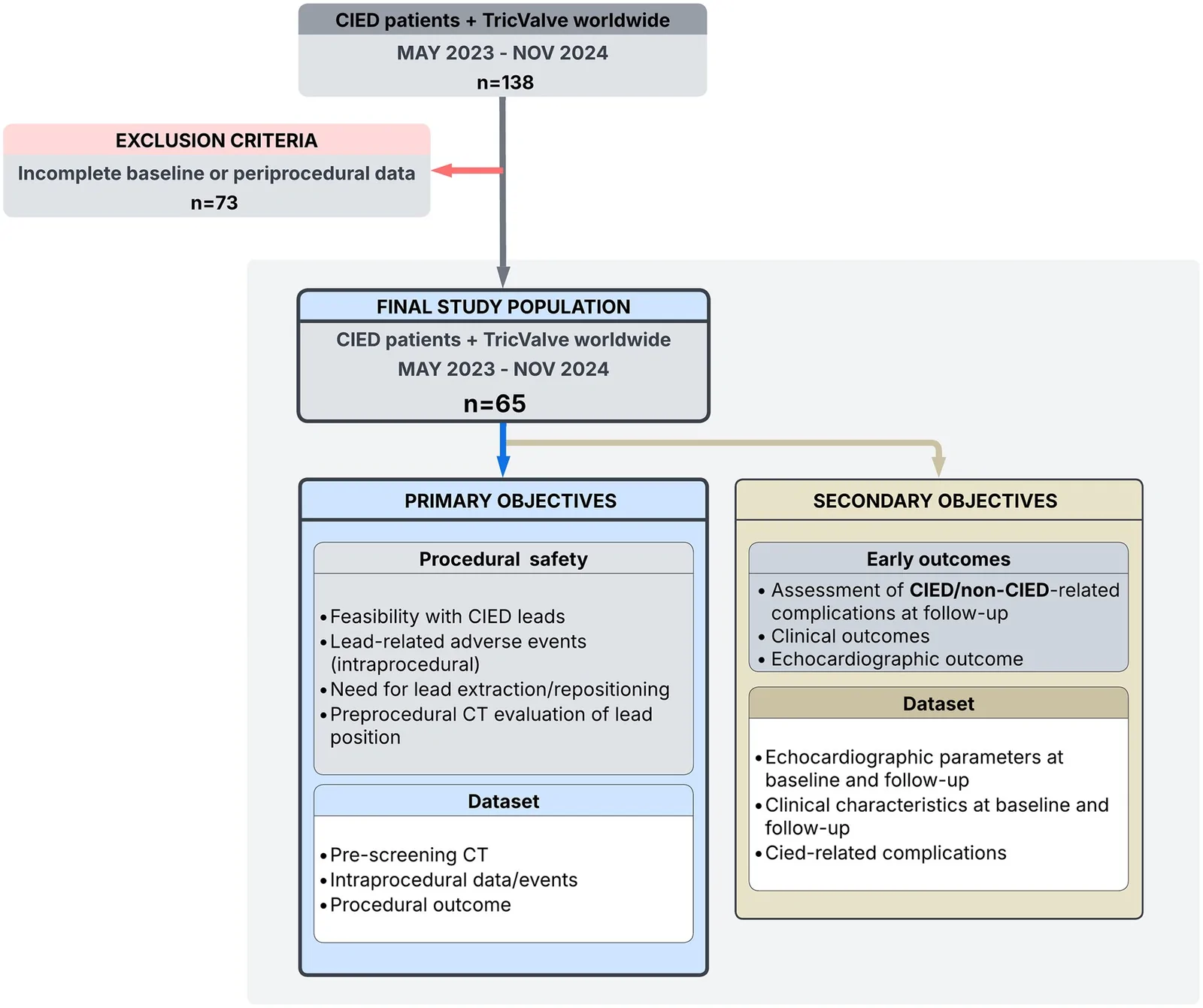
Study Design and Data Availability. Overview of the study population, primary and secondary objectives, and data availability. A total of 65 patients from 23 centres with preexisting cardiac implantable electronic devices (CIEDs) who underwent TricValve implantation between May 2023 and November 2024 were included in the procedural dataset. Abbreviation: CT, computed tomography.

This multicentre study was approved by the local ethics committee (EK no. 1996/2024). Participating centres contributed anonymized data in accordance with their local regulatory requirements. Given the retrospective design of the study and the use of fully anonymized data, the requirement for informed consent was waived.

### Lead position assessment with preprocedural CT imaging

To assess whether lead and electrode tip positions in the right atrium and right ventricle (RV) influenced procedural outcomes and the risk of lead-related complications, preprocedural CT imaging was requested from Products + Features GmbH and independently evaluated by the investigators using 3mensio Structural Heart (Pie Medical Imaging, Maastricht, the Netherlands). Computed tomography imaging of sufficient quality for analysis was available in 34 of 65 patients (52.3%) and was included in the final analysis.

Right atrial lead tip positions were classified as septal, free wall, or right atrial appendage (**Figure 2**). The free wall was further subdivided into low and mid free wall to provide a more detailed description of atrial lead position. Similarly, RV lead positions were categorized as septal, free wall, and apical positions.

**Figure 2.**
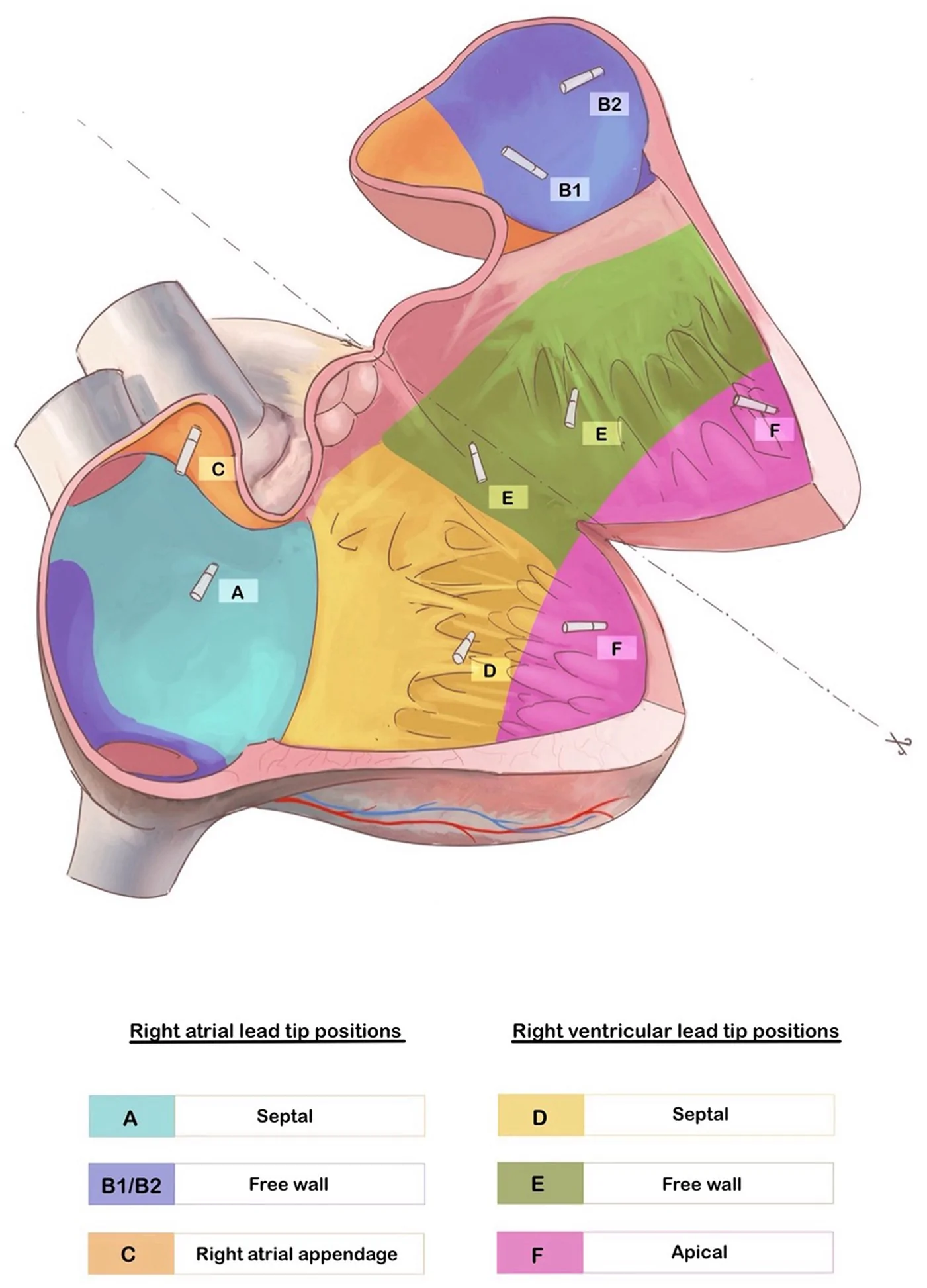
Schematic Illustration of Right Atrial and Right Ventricular Lead Tip Positions. Right atrial lead tip positions were classified as septal (A), free wall, or right atrial appendage (C). Free wall positions were further subdivided into mid free wall (B1) and low free wall (B2). Right ventricular lead tip positions were categorized as septal (D), free wall (E), or apical (F).

### Echocardiographic data analysis

As described above, 23 participating centres provided data for 65 patients, including echocardiographic examinations that were performed at 2 time points: before TricValve implantation and at the last available follow-up. Data collected comprised TR severity, aetiology of TR, left ventricular function, pulmonary artery pressure, and lead-valve interaction, with particular attention to lead impingement or perforation before and after the procedure. Tricuspid regurgitation severity was graded according to current recommendations using a 5-class scheme (mild, moderate, severe, massive, and torrential) as endorsed by recent consensus statements.^10–12^ Left ventricular ejection fraction (LVEF) was reported as a percentage. All echocardiographic analyses were performed at the participating centres and submitted in anonymized form for central analysis.

### Statistical analysis

All data were analysed descriptively. Continuous variables are reported as mean ± standard deviation (SD) or median with IQR, as appropriate. Categorical variables are summarized as absolute numbers and percentages. For the comparison of TR severity and LVEF between baseline and follow-up, the Wilcoxon signed-rank test was used. Statistical analyses were conducted using SPSS Statistics version 31 (IBM Corp.).

## Results

### Pre-procedural data

Baseline characteristics, echocardiographic findings, and device-related information of the 65 patients are summarized in **Tables 1-3**. The mean age was 78.1 ± 9.2 years (median 78 years, IQR: 75-84), and 52.3% were male (**Table 1**). The mean body mass index (BMI) was 25 kg/m² (IQR: 22-29). Most patients were in New York Heart Association (NYHA) functional class III (67.7%), and all patients presented with severe or greater TR (38.5% severe, 33.8% massive, and 27.7% torrential). The most frequent comorbidities were hypertension, atrial fibrillation (AF), and chronic kidney disease. Previous valve surgery had been performed in 20 patients (30.7%). In addition, 14 patients (21.2%) had undergone prior transcatheter valve interventions, including transcatheter tricuspid edge-to-edge repair (TriClip) in 7 patients (10.8%).

**Table 1.** Baseline Characteristics

Variables	All patients (*n *= 65)
Age (years), 25th-75th interval	78 (75-84)
Gender male, *n* (%)	34 (52.3%)
Body mass index, kg/m²	25 (22-29)
NYHA functional class, *n* (%)	
NYHA II:	12 (18.5%)
NYHA III:	44 (67.7%)
NYHA IV:	9 (13.8%)
Diabetes mellitus, *n* (%)	9 (13.8%)
Hypertension, *n* (%)	37 (56.9%)
Chronic kidney disease, *n* (%)	27 (41.5%)
Coronary artery disease, *n* (%)	20 (30.8%)
Peripheral artery disease, *n* (%)	2 (3.1%)
Cerebrovascular disease, *n* (%)	7 (10.8%)
History of stroke, *n* (%)	4 (6.2%)
Cardiomyopathy, *n* (%)	11 (16.9%)
Dilated	3 (4.6%)
Ischaemic	6 (9.2%)
Valvular	1 (1.5%)
Hypertrophic	1 (1.5%)
Atrial fibrillation, *n* (%)	52 (80.0%)
Previous valve surgery, *n* (%)	20 (30.7%)
Previous transcatheter valve	14 (21.5%)
Intervention, *n* (%)	
M-TEER only	4 (6.2%)
T-TEER only	5 (7.7%)
Mitral + tricuspid TEER	2 (3.1%)
TAVR	2 (3.1%)
TMVR	1 (1.5%)

Abbreviations: NYHA, New York Heart Association; TAVR, transcatheter aortic valve replacement; TEER, transcatheter edge-to-edge repair; TMVR, transcatheter mitral valve replacement.

**Table 2.** Device Data

Variables	All patients (*n* = 65)
Implanted cardiac devices, *n* (%)	
PPM	46 (70.8%)
ICD	6 (9.2%)
CRT-D	9 (13.8%)
CRT-P	3 (4.6%)
Not reported	1 (1.5%)
Device manufacturer, *n* (%)	
Medtronic	23 (35.4%)
Abbott/St. Jude Medical	15 (23.1%)
Biotronik	7 (10.7%)
Boston Scientific	4 (6.2%)
Microport	1 (1.5%)
Sorin	1 (1.5%)
Not reported	14 (21.5%)
Indication for implantation, *n* (%)^a^	
Sick sinus syndrome	13 (20.0%)
AV block (II or III)	23 (35.4%)
Brady-tachy syndrome	8 (12.3%)
Atrial fibrillation (any indication)	8 (12.3%)
Ventricular tachyarrhythmias (VT/VF)	4 (6.2%)
Other	10 (15.4%)
Not reported	3 (4.6%)
Implantation site, *n* (%)	
Left side	55 (84.6%)
Right side	8 (12.3%)
Both sides	1 (1.5%)
Not reported	1 (1.5%)

a Multiple indications possible. Abbreviations: AV, atrioventricular; CRT-D, cardiac resynchronization therapy-defibrillator; CRT-P, cardiac resynchronization therapy-pacemaker; ICD, implantable cardioverter defibrillator; PPM, permanent pacemaker; VF, ventricular fibrillation; VT, ventricular tachycardia.

**Table 3.** Baseline Echocardiographic Characteristics

Variables	All patients (*n *= 65)
Tricuspid regurgitation severity, *n* (%)	
Severe	25 (38.5%)
Massive	22 (33.8%)
Torrential	18 (27.7%)
Aetiology of tricuspid valve regurgitation, *n* (%)	
Primary	3 (4.6%)
Secondary	26 (40.0%)
Mixed	28 (43.1%)
With lead/device-related	24 (36.9%)
Lead-/device-related (isolated)	4 (6.2%)
Not reported	4 (6.2%)
Concomitant valvular disease, *n* (%)	
Aortic stenosis ≥ moderate	0 (0%)
Aortic regurgitation ≥ moderate	4 (6.2%)
Mitral stenosis ≥ moderate	2 (3.1%)
Mitral regurgitation ≥ moderate	24 (36.9%)
Pulmonary hypertension, *n* (%)	
None	8 (12.3%)
Mild (20-40 mmHg)	28 (43.1%)
Moderate (41-55 mmHg)	17 (26.2%)
Severe (>55 mmHg)	6 (9.2%)
Not reported	6 (9.2%)
Left ventricular ejection fraction, *n* (%)	
Median (IQR)	54.5% (50%-60%)
Preserved (≥50%)	47 (72.3%)
Mildly reduced (41%-49%)	10 (15.4%)
Reduced (≤40%)	8 (12.3%)
Lead position relative to the tricuspid valve, *n* (%)^a^	
Posteroseptal commissure	18 (27.7%)
Anteroseptal commissure	18 (27.7%)
Anteroposterior commissure	7 (10.8%)
Central position	15 (23.0%)
Posterior leaflet	4 (6.2%)
Septal leaflet	4 (6.2%)
Anterior leaflet	0 (0.0%)
Not reported	7 (10.8%)
Lead-tricuspid valve interaction, *n* (%)^a^	
Posterior impingement	15 (23.0%)
Septal impingement	15 (23.0%)
Anterior impingement	4 (6.2%)
No impingement	24 (36.9%)
Adherent (posterior/septal/anterior)	6 (9.2%)
Perforation (posterior/septal/anterior)	2 (3.1%)
Not reported	8 (12.3%)

a Percentages based on total cohort (*N* = 65); in patients with multiple leads, each position/interaction was counted separately.

Pacemakers were the most frequently implanted devices (70.8%), followed by cardiac resynchronization therapy (CRT) and implantable cardioverter defibrillator (ICD) systems (**Table 2**). The most common indications for device implantation were atrioventricular block and sick sinus syndrome (35.4% and 20.0%).

According to echocardiographic data (**Table 3**), the most frequent aetiology of TR was secondary and mixed, with 36.9% of cases being pacemaker (PM) related. The mean LVEF was 52.8 ± 9.8% (median 54.5%, IQR: 50-60). Pulmonary hypertension was present in 78.5% of patients, most frequently graded as mild (43.1%) or moderate (26.2%). With regard to lead position relative to the tricuspid valve, the most common locations were the posteroseptal and anteroseptal commissures (each 27.7%), followed by a central position in 23.0% of patients. Less frequent sites included the anteroposterior commissure (10.8%), posterior leaflet (6.2%), and septal leaflet (6.2%) (**Figure 3**). Lead impingement of the tricuspid valve was documented in 52.3% of cases, most frequently affecting the posterior (23.0%) or septal leaflet (23.0%), and less commonly the anterior leaflet (6.2%).

**Figure 3.**
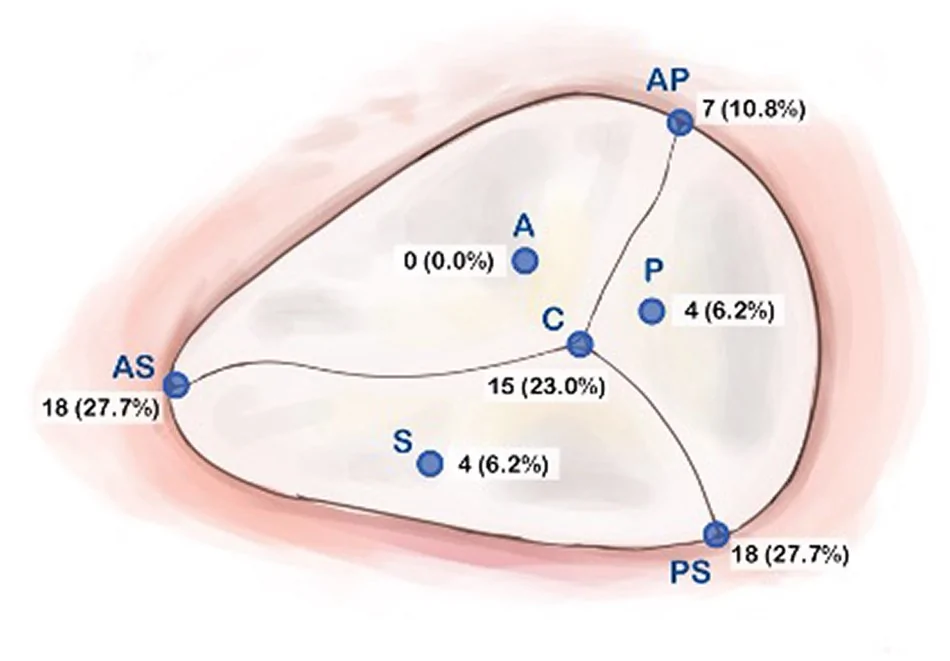
Lead Position Relative to the Tricuspid Valve. Lead positions relative to the tricuspid valve are shown as posteroseptal commissure (PS), anteroseptal commissure (AS), anteroposterior commissure (AP), central position (C), posterior leaflet (P), septal leaflet (S), and anterior leaflet (A). Values are presented as *n* (%).

### Device lead position before TricValve implantation

**Figure 4** demonstrates the percentage distribution of lead tip positions in the right atrium and RV, as assessed by preprocedural CT imaging in 34 patients. The most frequent right atrial lead tip position was in the right atrial appendage (13 lead tips of 20 atrial leads, 65.0%). The second most common location was along the free wall (3 lead tips in mid free wall [15.0%] and 3 in low free wall [15.0%]), whereas septal positioning was observed only rarely (1 lead tip, 5.0%). In cases of septal or free wall positioning, some patients were noted to have the lead tip located in close proximity to the tricuspid valve. For leads positioned in the right atrial appendage, some patients had very short leads with the tip located anatomically near the superior cavoatrial junction, a finding that potentially increased the risk of lead dislocation during intervention.

**Figure 4.**
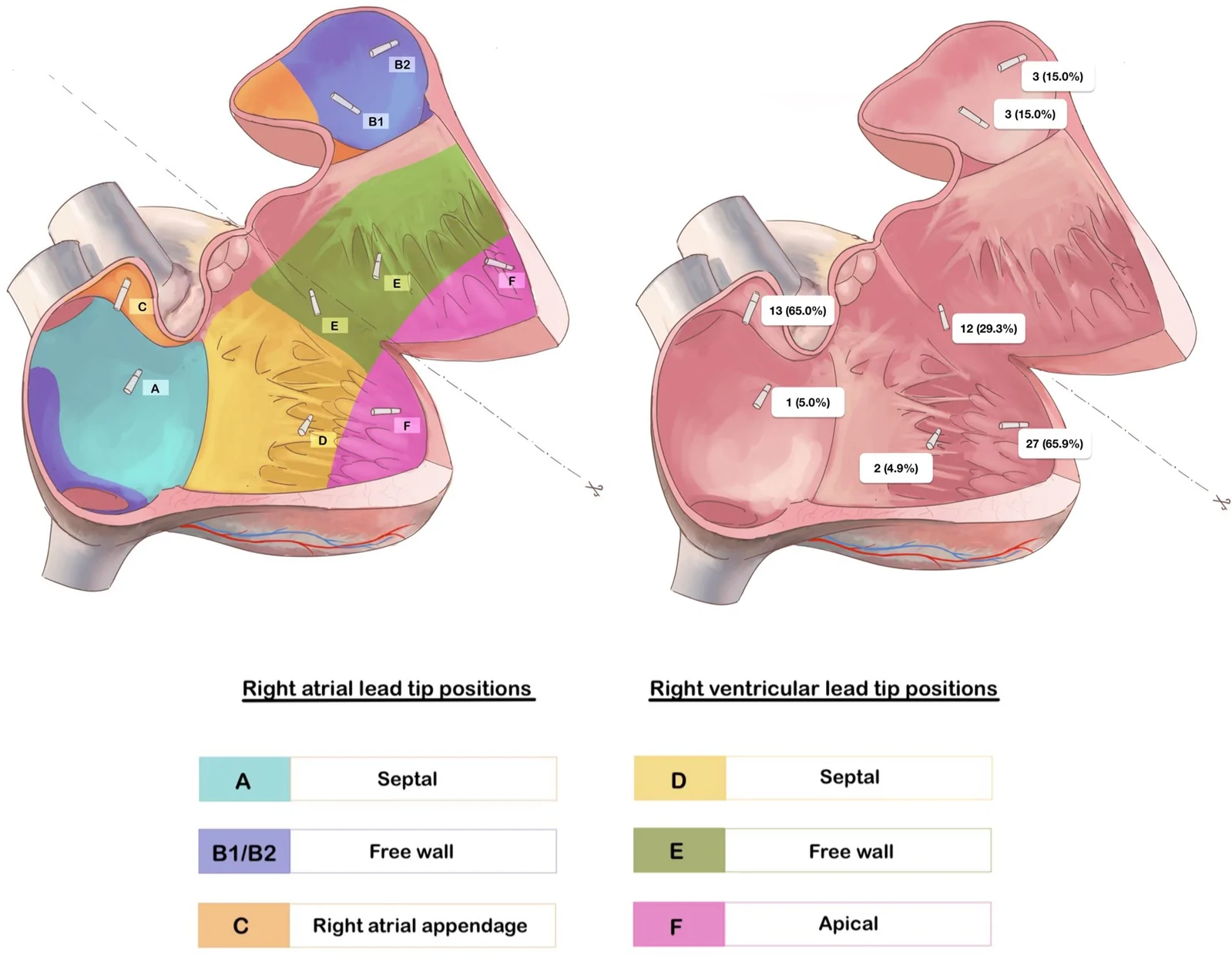
Distribution of Right Atrial and Right Ventricular Lead Tip Positions. Percentage distribution of lead tip positions in the right atrium (20 leads) and right ventricle (41 leads) assessed by pre-procedural computed tomography in 34 patients. Right atrial leads were most frequently located in the right atrial appendage (65.0%), followed by the free wall (mid 15.0%, low 15.0%) and septum (5.0%). Right ventricular leads were predominantly apical (65.9%), with fewer free wall (29.3%) and septal positions (4.9%).

The most frequent lead tip position in the RV was apical (65.9%). In 29.3% of all cases, the lead tip was positioned on the RV free wall. In 3 of these patients, the lead tip was located very close to the RV outflow tract. Septal positioning was rare (2 patients, 4.9%), with both leads located near the tricuspid valve.

In most cases, only a single lead was positioned in the RV, whereas in 5 patients, 2 or 3 leads were present. In these cases, the lead tips were either both located apically or distributed between the apex and free wall positions.

Regarding the course of the leads themselves, most were freely positioned without direct contact with the right atrial walls. In 10 patients, however, the course was different, with longer leads running along the floor of the right atrium just above or close to the inferior cavoatrial junction (**Figure 5A**). In cases where the lead coursed along the atrial floor, its trajectory continued across the tricuspid valve to the RV floor. This configuration was associated with posterior leaflet impingement or adhesion in selected patients. In 1 case, a very long RV lead formed a large loop in the right atrium, which partially interacted with the tricuspid valve (**Figure 5B**).

**Figure 5.**
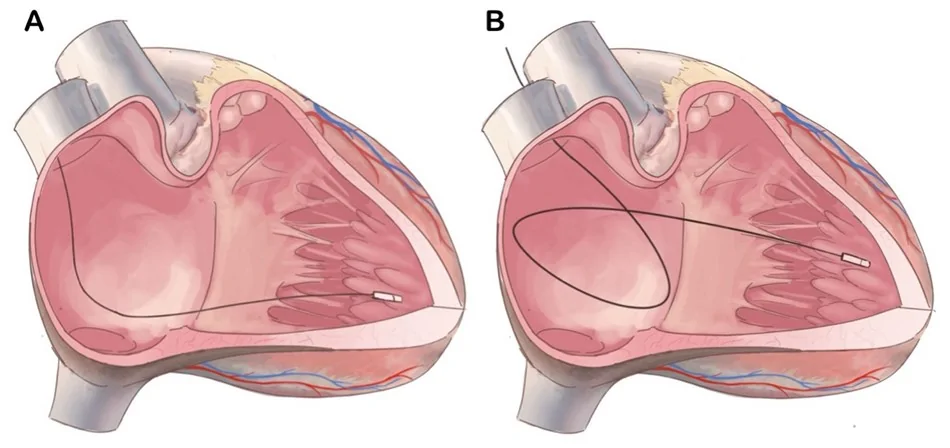
Atypical Lead Courses in the Right Atrium. (A). In selected patients, longer leads course along the floor of the right atrium just above or close to the inferior cavoatrial junction, with continuation across the tricuspid valve towards the right ventricular floor, a configuration associated with posterior leaflet impingement or adhesion. (B). Example of a very long right ventricular lead forming a large loop within the right atrium with partial interaction with the tricuspid valve.

Seven patients presented with 1, 2, or 3 TriClips with RV leads following different courses in proximity to the clips.

### Procedural and in-hospital outcome

**Table 4** summarizes detailed procedural and in-hospital outcomes. Device implantation was successful in all cases (100%). Despite the presence of PM or CRT leads, no lead extractions were required or performed prior to the intervention.

**Table 4.** Procedural Characteristics and In-hospital Outcomes

Variables	All patients (*n *= 65)
Device successfully implanted, *n* (%)	65 (100%)
CIED-associated complications, *n* (%)	4 (6.2%)
Other procedural complications, *n* (%)	
Femoral access-site bleeding	4 (6.2%)
Shoulder pain	4 (6.2%)
Pneumonia	2 (3.1%)
Urinary tract infection	3 (4.6%)
Heart failure exacerbation	3 (4.6%)
Acute kidney injury	2 (3.1%)
In-hospital mortality, *n* (%)	4 (6.2%)

Abbreviation: CIED, cardiac implantable electronic devices.

Overall, 4 CIED-related complications (6.2%) were reported. Intraprocedural atrial lead dislocation occurred in 1 case. During the index hospitalization, PM reprogramming was also required in 1 case. In 2 other cases, PM function ceased after the intervention for unknown reasons, although no lead dislocation was observed. In the remaining patients (93.8%), no PM dysfunction was observed after the procedure.

Beyond CIED-related outcomes, other periprocedural complications were reported; these included femoral access-site bleeding in 4 patients (6.2%), shoulder pain in 4 (6.2%), pneumonia in 2 (3.1%), and urinary tract infection in 3 (4.6%) cases. Three patients developed heart failure exacerbation (4.6%), and 2 patients developed acute kidney injury (3.1%). Four patients (6.2%) died during the index hospitalization: 1 due to multiple organ dysfunction syndrome (MODS) and 3 (4.6%) due to combined respiratory and cardiac decompensation.

### Follow-up outcomes

Clinical and echocardiographic data (**Tables 5** and **6**, respectively) were available for all 65 patients, with a median follow-up of 159 days (IQR 60-358 days). Thirty-day mortality was 6.2% and consisted entirely of the in-hospital deaths reported above. Beyond 30 days, 3 additional deaths occurred during follow-up (4.6%). Two patients died within 7 months after the procedure, 1 from respiratory failure and 1 from myocardial infarction, whereas the third patient died 3 months after the procedure due to refractory heart failure. Overall, all-cause mortality during the entire follow-up period was 10.8%.

**Table 5.** Clinical Outcomes at Follow-up

Variables	All patients (*n *= 65)
Device-related complications, *n* (%)^a^	0 (0.0%)
New lead-valve interaction, *n* (%)^b^	0 (0.0%)
Non-device-related complications, *n* (%)	2 (3.1%)
Endocarditis	1 (1.5%)
Paravalvular leak	1 (1.5%)
30-day mortality, *n* (%)	4 (6.2%)
All-cause mortality during follow-up, *n* (%)	7 (10.8%)

a Includes PM/CRT/lead replacement or reimplantation.

b Includes lead dislodgement, adhesions, migration, and perforation.

**Table 6.** Changes in TR Severity and LV Function Before and After TricValve Implantation

Variables	Baseline, *n* (%)	Follow-up, *n* (%)	*P* value^a^
Tricuspid regurgitation severity, *n* (%)			.009
Mild	0 (0.0%)	3 (4.6%)	
Moderate	0 (0.0%)	2 (3.1%)	
Severe	26 (40.0%)	29 (44.6%)	
Massive	22 (33.8%)	16 (24.6%)	
Torrential	17 (26.2%)	11 (16.9%)	
Not reported^b^	0 (0.0%)	4 (6.2%)	
LVEF, % (median [IQR])	54 (50-60)	55 (49-60)	.766
Preserved (≥50%)	50 (76.9%)	45 (69.2%)	
Mildly reduced (41%-49%)	7 (10.8%)	8 (12.3%)	
Reduced (≤40%)	8 (12.3%)	7 (10.8%)	
Not reported^b^	0 (0.0%)	5 (7.7%)	

a Wilcoxon signed-rank test.

b Patients with missing data were excluded from paired statistical analysis. Abbreviation: LVEF, left ventricular ejection fraction.

One patient developed endocarditis with an 8 × 7 mm vegetation on the inferior vena cava (IVC) portion of the TricValve system, which was successfully treated with antibiotics, with no residual vegetations detectable after 7 weeks. In another case, transthoracic echocardiography performed 3 months after the index procedure revealed a small paravalvular leak at the IVC prosthesis. No other complications were detected (**Table 5**).

Tricuspid regurgitation severity significantly improved after TricValve implantation (*P* = .009, Wilcoxon signed-rank test) (**Table 6**). Improvement was observed in 20 patients (30.8%), worsening in 7 (10.8%), and no change in 34 (52.3%). Left ventricular ejection fraction remained unchanged after TricValve implantation (baseline 54% [IQR: 50-60] vs follow-up 55% [IQR: 49-60]; *P* = .77, Wilcoxon signed-rank test).

Regarding cardiac rhythm, 3 patients in sinus rhythm prior to the procedure remained in sinus rhythm at follow-up; additionally, 2 patients with a history of paroxysmal AF were in sinus rhythm at follow-up. No rhythm changes were observed in the remaining patients. In all 34 cases with known impinging leads before TricValve implantation, findings remained unchanged at follow-up. No additional lead-related complications were observed during the follow-up period.

## Discussion

Our findings are consistent with prior reports demonstrating the safety of TricValve implantation in patients with severe TR who are not suitable for surgical valve repair or replacement, or in whom other transcatheter interventions were anatomically not feasible or had previously failed to provide clinical improvement. However, previous studies have not specifically addressed an important subgroup of patients—those with preexisting CIEDs. In this population, device leads may represent an additional risk and could complicate TricValve implantation. The key clinical questions were whether implantation is technically feasible in the presence of leads, whether lead dislocation may occur during the procedure, leading to device malfunction or damage to the tricuspid valve, and whether pre-implantation lead extraction should be considered.

To date, no published data have systematically evaluated these questions or the potential lead-related risks specifically associated with bicaval valve implantation using the TricValve system. However, several studies have already reported outcomes of EVOQUE TTVR in the presence of “jailed” leads, providing valuable insight into the types and frequency of lead-related complications that may also be relevant when considering intervention with the TricValve system.

According to the TRISCEND II trial (Transcatheter Tricuspid Valve Replacement: Pivotal Clinical Investigation of Safety and Clinical Efficacy Using a Novel Device), and after excluding certain high-risk CIED scenarios—such as PM-dependent patients without an alternative pacing strategy—approximately 38% of patients who underwent TTVR had a CIED, representing nearly one-third of all patients enrolled in the study. Unfortunately, neither TRISCEND I nor TRISCEND II specifically reported outcomes in patients with preexisting CIEDs. However, data presented by Garcia at the TCT (Transcatheter Cardiovascular Therapeutics) 2025 conference in San Francisco, based on the combined TRISCEND I and II patient cohorts, showed that among the 152 patients with a preexisting CIED included in the analysis, 10 patients (6%) developed post-procedural lead-related complications. According to their report, lead replacement or repositioning was required in 6 cases, 2 cases were managed by adjustments in device programming, and 2 additional cases were treated conservatively.^13^

In another study by Braunstein et al,^14^ among 21 patients with preexisting CIED leads, only 1 patient experienced an acute lead dislodgment during the TTVR procedure, which required implantation of a leadless PM. One additional patient developed a self-limiting, small acute increase in pacing threshold.

A similar outcome with a low complication rate was also reported in a study by Anderson et al,^15^ in which only 1 case (4%) of RV lead dislodgment occurred among 28 patients with an entrapped RV lead. In the study by Peigh et al,^16^ a total of 4 lead-related complications were documented among 52 patients (7.7%), including 1 case of methicillin-resistant *Staphylococcus aureus* (MRSA) endocarditis involving the “jailed” lead system occurring 1 month after TTVR.

Notably, the study by Abbasi et al reported a substantially higher incidence of post-procedural lead abnormalities following TTVR with the EVOQUE system, highlighting that earlier reports may have underestimated lead-related complications.^17^^,^^18^ In their cohort of 32 patients with median follow-up of 210 days, approximately 31% exhibited device-lead abnormalities, including changes in sensing or pacing parameters. Although many of these findings were managed conservatively and did not require immediate intervention, 4 patients demonstrated lead insulation breaches, and 1 patient required lead revision. The study by Mekary et al^19^ also reported severe lead dysfunction in 3 of 14 patients with jailed leads (20%), including fatal complication following extraction of a jailed lead.

In contrast, in our TricValve cohort, clinically relevant post-interventional lead-related complications were observed in only 6.2% of 65 patients, substantially lower than the rates reported by Abbasi et al and other studies.

In the first case described by Mi Chen et al, TricValve implantation was performed off-label because preprocedural screening revealed an extremely small and short SVC, far below reference values. During advancement of the SVC delivery system, PM leads were displaced into the right brachiocephalic vein, creating an obstruction that required snaring and traction to reposition them. These maneuvers caused atrial lead dislocation, which was corrected after successful deployment of the SVC and IVC valves.^20^

In another case, post-procedural PM interrogation revealed atrial lead dysfunction, characterized by absent atrial sensing and loss of capture. Chest radiography excluded lead dislocation. The device was maintained in the already programmed VVIR mode for AF, and a conservative management strategy was adopted. The dysfunction was therefore considered potentially related to mechanical damage to the atrial lead during TricValve implantation.

In the third case, PM function ceased after implantation for unclear reasons, necessitating device replacement. In the fourth case, PM reprogramming was required during the index hospitalization, after which adequate function was restored. Although direct mechanical interaction or compression of the pacing lead by the SVC prosthesis cannot be excluded, no objective evidence was available to support these mechanisms in the affected patients.

Comparable to prior EVOQUE TTVR studies, our results indicate a lower overall complication rate; nonetheless, lead-related events remain possible with both TTVR and TricValve interventions, and prior studies have not systematically characterized risk factors for lead dislodgement or other device-related complications. Moreover, there are currently no evidence-based criteria or guideline recommendations that define which patients with CIEDs are at increased risk of clinically significant lead dislodgement and might therefore benefit from prophylactic lead extraction prior to intervention.

In the published case-review series by Guddeti et al, the authors discuss strategies for managing CIEDs during TTVR and emphasize that the risks and benefits of TLE should be carefully considered—particularly in patients with CIED-induced TR or in PM-dependent individuals treated at high-volume extraction centres if procedural risk is low. In such scenarios, alternative pacing strategies, including leadless PM implantation, coronary-sinus pacing, or epicardial lead placement, may be required. The authors also recommend proceeding with TTVR without lead removal in non- PM-dependent patients, especially when the RV lead is positioned in a commissural location and does not interfere with valve deployment.^21^

In an editorial by Defaye et al,^17^ the authors caution that post-interventional lead extraction following TTVR—particularly in the presence of an implanted EVOQUE valve—may carry a risk of valve destabilization or migration.

In the context of TricValve implantation in the presence of PM leads, such issues were not observed; moreover, some potential lead dislocations may be avoided by device recapture and repositioning. During TricValve implantation in the SVC, particular caution is required in the presence of a preexisting PM lead, when the lead is short (**Figure 6**). In cases where the lead becomes jailed against the lateral wall of the SVC during partial deployment of the prosthesis, there is a risk of lead dislocation (**Figure 6A**). To avoid this complication, the valve may be recaptured and repositioned. A controlled, slow deployment is recommended to ensure that the lead is jailed along the medial wall of the SVC, thereby reducing the risk of lead dislodgement (**Figure 6B**).

**Figure 6.**
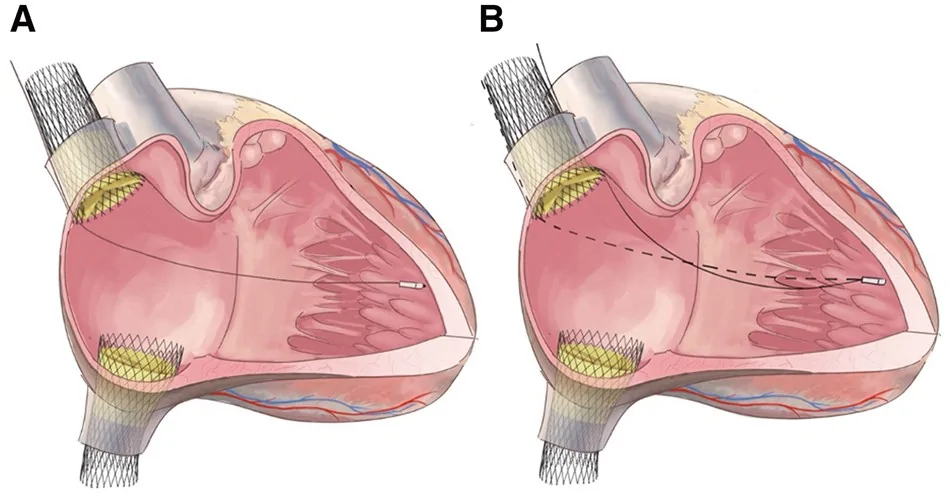
Pacemaker Lead-Prosthesis Interaction During TricValve Implantation. (A). Partial deployment of the TricValve prosthesis in the superior vena cava with jailing of a short preexisting pacemaker lead against the lateral wall, a configuration associated with risk of lead dislocation. (B). Controlled deployment with recapture and repositioning of the prosthesis, resulting in jailing of the pacemaker lead along the medial wall of the superior vena cava, thereby minimizing the risk of lead dislocation.

An additional consideration is the potential challenge of future TLE in patients with a lead jailed within the SVC. Should device infection or lead failure occur, extraction may become technically challenging and carry additional risks. However, there are currently no data on the long-term incidence of these scenarios following TricValve implantation. Consequently, it remains unknown whether this potential downstream risk justifies routine prophylactic lead extraction before TricValve implantation, particularly in view of the established procedural risks associated with TLE. In our cohort, 1 patient developed MRSA endocarditis involving the jailed lead system 1 month after TricValve implantation. The infection was successfully treated with antibiotic therapy, with complete resolution after 7 weeks, and no lead extraction was required.

Taken together, our results support the feasibility and safety of TricValve implantation in patients with preexisting PM or CRT systems. Among the 65 patients with available data for CIED-associated complications, only 4 (6.2%) experienced lead-related complications. Notably, all these events were manageable, and no TricValve malfunctions or valve damage were observed. No reoperation or untreatable endocarditis was reported. These findings suggest that although lead-related issues may occur, they should not be considered a contraindication to TricValve implantation in patients with preexisting PM or CRT systems. Importantly, routine lead extraction prior to TricValve implantation may significantly increase procedural risk due to potentially severe complications such as cardiac or vascular perforation, tamponade, tricuspid valve damage, pulmonary embolism, infection, and loss of pacing in PM-dependent patients. No cases of life-threatening device malfunction were observed, and mortality was not related to pacing complications, confirming the safety of TricValve system use in this patient population. Longer-term follow-up is required and will be performed to clarify long-term adverse event rates.

This study has several limitations. First, as a retrospective analysis, it is subject to potential selection bias, missing data, and variability in data collection across centres. Although interobserver variability may have influenced some secondary imaging assessments, it is unlikely to have affected the interpretation of clinically relevant lead-related events, which represented the primary study objective. Second, as TricValve implantation is a relatively recent therapy, follow-up was inherently limited to the maximum duration currently available, with a median follow-up of 159 days (IQR 60-358). Consequently, longer-term clinical outcomes cannot yet be fully assessed; however, extended follow-up data will become available over time and will allow further evaluation.

Another limitation is that detailed quantitative CIED interrogation data (eg pacing thresholds, sensing amplitudes, and lead impedance) as well as minor changes in device function were not systematically collected. Given the retrospective multicentre design involving numerous international centres with heterogeneous follow-up protocols, standardized assessment of these parameters was not feasible and would likely have resulted in substantial missing and inconsistent data. Moreover, these parameters were beyond the predefined objectives of the present study, which focused on clinically relevant lead-related complications and the overall feasibility and safety of TricValve implantation.

Despite these limitations, the present study represents the first international multicentre observational study to systematically evaluate TricValve implantation in patients with preexisting CIEDs using complete datasets for the predefined primary study objectives. By including a substantial proportion of all patients with CIEDs treated with the TricValve system worldwide during the study period, our findings provide the first robust real-world evidence on the feasibility and short-term safety of this approach without routine lead extraction or lead repositioning. We believe these data address an important knowledge gap and provide a valuable foundation for future prospective studies with standardized device interrogation and longer-term follow-up.

## Conclusion

Our study supports the feasibility of TricValve implantation in patients with preexisting CIEDs, with a low rate of complications observed during early follow-up. Lead-related complications were rare, manageable, and did not compromise device function or procedural success. Our data do not support routine prophylactic lead extraction before TricValve implantation; however, individual anatomical characteristics and clinical judgement remain essential when planning the optimal treatment strategy. Larger prospective studies with longer follow-up will be of value to validate these findings and to define strategies for managing patients with complex lead anatomies.

## Data Availability

Data are available in a repository and can be accessed via a DOI link.

## Abbreviations

AF: Atrial fibrillation
BMI: Body mass index
CIED: Cardiac implantable electronic device
CRT: Cardiac resynchronization therapy
CT: Computed tomography
ICD: Implantable cardioverter defibrillator
IQR: Interquartile range
IVC: Inferior vena cava
LVEF: Left ventricular ejection fraction
MODS: Multiple organ dysfunction syndrome
MRSA: Methicillin-resistant *Staphylococcus aureus*
NYHA: New York Heart Association
PM: Pacemaker
RV: Right ventricle/right ventricular
SD: Standard deviation
SVC: Superior vena cava
TEE: Transesophageal echocardiography
TR: Tricuspid regurgitation
TTE: Transthoracic echocardiography
TTVR: Transcatheter tricuspid valve replacement
TLE: Transvenous lead extraction
